# Scoping review on the role of the family doctor in the prevention and care of patients with foetal alcohol spectrum disorder

**DOI:** 10.1186/s12875-024-02291-x

**Published:** 2024-02-22

**Authors:** Sébastien Leruste, Bérénice Doray, Thierry Maillard, Christophe Lebon, Catherine Marimoutou, Michel Spodenkiewicz

**Affiliations:** 1 INSERM CIC-EC 1410, CHU de La Réunion, BP350 - 97 448, Saint-Pierre Cedex, La Réunion, France; 2 UFR Santé, University of La Réunion, 97410 Saint-Pierre, France; 3 Service de Génétique - CHU de La Réunion, Saint-Denis, France; 4 Laboratoire EPI, Université & CHU de La Réunion, Saint-Denis, France; 5 Centre Ressources, TSAF - Fondation Père Favron - CHU de La Réunion, Saint-Pierre, France; 6 SAF-Ocean India, Saint-Louis, France; 7 Moods Team, INSERM UMR-1178, CESP, Le Kremlin-Bicêtre, France; 8 McGill Group for Suicide Studies, Department of Psychiatry, Douglas Mental Health University Institute, McGill University, Montréal, Canada

**Keywords:** Family doctor, Foetal alcohol spectrum disorder, Prevention, Care, Scoping review

## Abstract

**Background:**

Foetal alcohol spectrum disorder (FASD) is the leading preventable cause of nongenetic mental disability. Given the patient care pathway, the General Practitioner (GP) is in the front line of prevention and identification of FASD. Acknowledging the importance of the prevalence of FASD, general practitioners are in the front line both for the detection and diagnosis of FASD and for the message of prevention to women of childbearing age as well as for the follow-up.

**Objectives:**

The main objective of the scoping review was to propose a reference for interventions that can be implemented by a GP with women of childbearing age, their partners and patients with FASD. The final aim of this review is to contribute to the improvement of knowledge and quality of care of patients with FASD.

**Methods:**

A scoping review was performed using databases of peer-reviewed articles following PRISMA guidelines. The search strategy was based on the selection and consultation of articles on five digital resources. The advanced search of these publications was established using the keywords for different variations of FASD: "fetal alcohol syndrome," "fetal alcohol spectrum disorder," "general medicine," "primary care," "primary care"; searched in French and English.

**Results:**

Twenty-three articles meeting the search criteria were selected. The interventions of GPs in the management of patients with FASD are multiple: prevention, identification, diagnosis, follow-up, education, and the role of coordinator for patients, their families, and pregnant women and their partners. FASD seems still underdiagnosed.

**Conclusion:**

The interventions of GPs in the management of patients with FASD are comprehensive: prevention, identification, diagnosis, follow-up, education, and the role of coordinator for patients, their families, and pregnant women and their partners.

Prevention interventions would decrease the incidence of FASD, thereby reducing the incidence of mental retardation, developmental delays, and social, educational and legal issues.

A further study with a cluster randomized trial with a group of primary care practitioners trained in screening for alcohol use during pregnancy would be useful to measure the impact of training on the alcohol use of women of childbearing age and on the clinical status of their children.

**Supplementary Information:**

The online version contains supplementary material available at 10.1186/s12875-024-02291-x.

## Background

The harmful effects of alcohol consumption in the prenatal period have been recognized and studied for half a century [1, 2]. Prenatal alcohol exposure causes a range of disorders of varying severity, including prematurity, intrauterine growth retardation, fetal loss and stillbirth [3].

The most comprehensive category is Fetal Alcohol Syndrome, which includes growth retardation, craniofacial dysmorphia and neurodevelopmental disorders.

Another category is Alcohol-Related Neurodevelopmental Disorder (ARND), which manifests itself in psycho-affective and socialization disorders, with difficulties in social interaction, and adjustment disorders linked to problems of memory, attention or hyperactive behavior.

Finally, the last category includes alcohol-related birth defects (ARBD), such as cardiac and musculoskeletal malformations, complete [4].

Of all the categories that make up FASD, ARND is the most common, and FAS probably represents only 5–7% of FASD.

Fetal alcohol syndrome (FAS) is the first described and best known FASD. It is sometimes considered the most severe form [5]. FAS is the leading cause of congenital nongenetic mental disability and social maladjustment in children. It is entirely preventable [6, 7]. It is the only form of FASD with an International Classification of Diseases (ICD) code: Q86.0 in the ICD-10 and LD2F.00 in the ICD-11 [8]. The diagnosis is primarily clinical (Table 1).

**Table 1:** Diagnostic criteria for Fetal Alcohol Syndrome

1) Confirmation of Prenatal Alcohol Exposure (PAE)
2) The characteristic craniofacial dysmorphia of FAS including:
- Narrow palpebral clefts,
- An elongated, bulging philtrum with no relief,
- A short nose with anteverted nostrils,
- A thin and narrow upper lip,
- A small recessed chin
3) Growth retardation either prenatal (intrauterine growth retardation (IUGR)) or postnatal. This delay affects head circumference, weight and height
4) Evidence of central nervous system (CNS) damage, which may be:
- structural: cerebral growth deficiency (e.g. microcephaly, agenesis of the corpus callosum, cerebellar hypoplasia)
- functional: manifested by mild or profound neurological disorders (depending on age) such as fine motor disorders, sensorineural deafness, poor gait, poor hand–eye coordination
These last three criteria (2, 3 and 4) constitute the symptomatic triad of the syndrome
Thus, when alcohol consumption is not documented but these three criteria are evident, the diagnosis of "FAS without confirmation of Prenatal Alcohol Exposure" can be made
5) The presence of behavioral and cognitive abnormalities that are inconsistent with developmental level and cannot be explained by family history or environment alone, such as learning disabilities, deficits in academic performance, poor control of impulsivity, difficulties in social perception, deficits in receptive and expressive language, reduced capacity for abstraction or metacognition, specific deficits in mathematics, problems with memory, attention, or judgment

Patients with FAS present a characteristic dysmorphic feature because maternal alcohol use during the first trimester of pregnancy disrupts normal brain and facial development of the fetus.

There are also partial forms of FAS, resulting in learning difficulties and/or impaired social adaptation (school failure, conduct disorders, delinquency, incarceration, marginality and substance abuse in adolescence) [9].

The generic term FASD is used to group all these symptomatic forms in which prenatal alcohol exposure is the primary aetiology.

Confirmation of prenatal alcohol exposure is necessary for the diagnosis of FASD.

"The diagnosis of FASD without FAS thus remains a syndromic diagnosis that associates proven and symptomatic neurocognitive deficits with prenatal alcohol exposure (PAE) in the absence of other detectable neurodevelopmental diseases" [10].

The severity of FASD is linked to the level of brain damage.

The Institute of Medicine (IOM) and Diagnostic and Statistical Manual of Mental Disorders V (DSM-5) terms are not listed as diagnostic conditions in the defined criteria, although they are useful for classification [11] (Table 2).

**Table 2:** FASD subtypes established by the Institute of Medicine in 1996)

Fetal Alcohol Syndrome (FAS)
Partial Fetal Alcohol Syndrome (pFAS)
Alcohol-Related Neurodevelopmental Disorder (ARND)
Alcohol-Related Birth Defects (ARBD)

The DSM–5 includes the Neurobehavioral Disorder Associated with Prenatal Alcohol Exposure (ND-PAE) regardless of the presence or absence of the physical effects of prenatal alcohol exposure. People who meet the criteria for an FASD diagnosis according to the IOM may also meet the criteria for ND-PAE. Thus, the neurodevelopmental deficits encountered in FASD are not pathognomonic [12]. However, diagnostic guidelines and a 4-digit code have been established that emphasize neurodevelopmental assessment [13–15]. The prevalence of FASD is 7.7‰ of births worldwide, with an extrapolation for France of 7,700 cases of births per year. ARND is ten times more common than FAS [16].

FASD is a common medical situation, included in primary care. However FASD remained largely underdiagnosed. Inadequate diagnostic practices might also play a role in prevalence and incidence rates, including miscarriages or stillbirths due to prenatal alcohol consumption [17].

Acknowledging the importance of the prevalence of FASD, general practitioners are in the front line both for the detection and diagnosis of FASD and for the message of prevention to women of childbearing age [18, 19] as well as for the follow-up [20–24]. In contrast to another form of neurodevelopmental disorder, such as autism spectrum disorder, articles published in peer-reviewed journals related to the prevention actions of FASD by the general practitioner have not yet been included in a scoping review [25].

The main objective of the literature review was to identify interventions that can be implemented by a general practitioner with women of childbearing age, their partners and patients with FASD.

## Methods

A scoping review was performed using databases of peer-reviewed articles following PRISMA guidelines for writing and reading systematic reviews and meta-analyses [26]

The search strategy was based on the selection and consultation of articles on the digital resources PsycINFO, Medline, PubMed and Cairn, complemented by a search in Google Scholar. The advanced search of these publications was established using the keywords for different variations of FASD: "fetal alcohol syndrome”, "fetal alcohol spectrum disorder”, “family medicine”, "general medicine", "primary care" or "primary care" searched in French and English.

### Inclusion criteria:

- Articles concerning family medicine, general medicine or primary care published in peer-reviewed journals
- Articles concerning FAS or FASD
- Articles in French or English
- No publication deadline

### Noninclusion criteria:

- Gray literature (i.e.: books, websites)

**Fig. 1:**
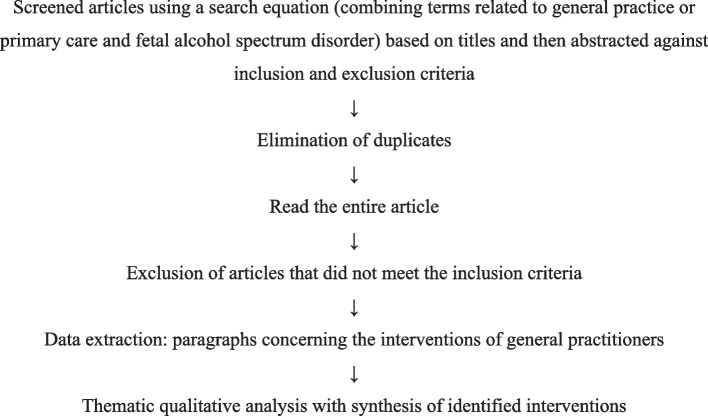
Flow chart of the article selection strategy

The selection process is summarized in Fig. 1.

The last search date was March 9, 2022.

SL reviewed all article titles and abstracts and selected those eligible for full-text review. MS checked the eligibility of full-text articles. SL and MS discussed the discrepancies to arrive at the final list. Articles were categorized according to the chosen plan for this work: prevention, identification/screening, diagnosis, and management of patients with fetal alcohol spectrum disorder. SL created a data collection form and tested it on three articles.

Their analysis was conducted using unstructured thematic qualitative analysis.

## Results

Twenty-three articles meeting the search criteria were selected.

**Fig. 2:**
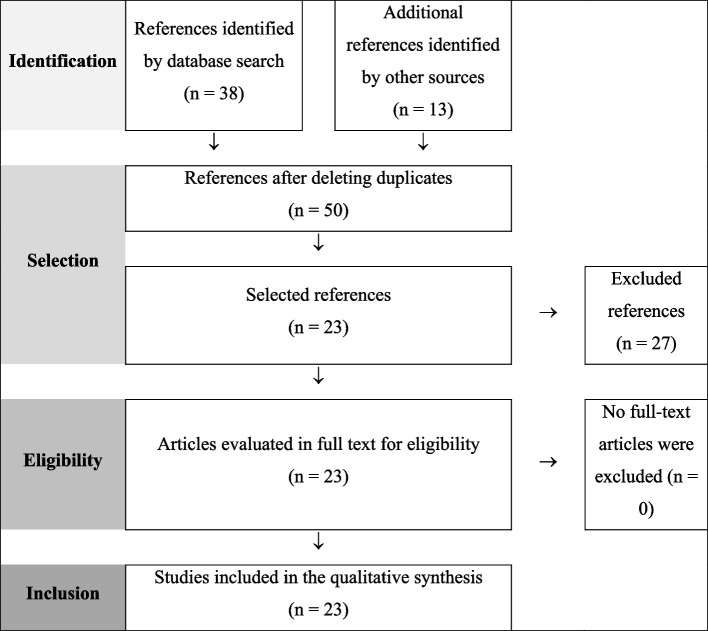
Flow diagram for the scoping review process adapted from PRISMA recommendations

The result of the scoping review process are summarized in Fig. 2.

The results of this scoping review are presented in a tabular form, summarizing the main findings (Table 3). They were further classified into subgroups according to their main theme: prevention, detection-screening, diagnosis, intervention, and therapeutics.

**Table 3:** Description of included articles

	#	AUTHOR	COUNTRY	TYPE OF ARTICLE	MAIN RESULTS
PREVENTION	[20]	Masotti et Al. 2003	Canada	Letter to the editor	Influential role of family in alcohol use among American adolescents Importance of considering multi-factorial elements (holistic, socio-economic, communication, family, notion of non-guilt) Approach must be culturally appropriate and accessible for Aboriginal women Primary role of the doctor/patient relationship (communication) Acceptable to clinicians and easily implemented
	[18]	Loock et al. 2005	Canada	Expert recommendations	Primary care physicians have a key role in preventing FASD in heavy drinkers (any woman with > 7 drinks/week or 3 drinks/day)
	[27]	Ramussen et al. 2010	Canada	Retrospective study	Evaluation of a prevention program for women at risk of having a child with FASD and significant pre/post program improvement in at risk birth control
	[21]	Zoorob et al. 2014	USA	Review of the literature	The Centers for Disease Control and Prevention's (CDC), the American Academy of Family Physicians (AAFP), and the American Congress of Obstetrics and Gynecology (ACOG) recommend that all women of childbearing age be targeted regarding alcohol consumption to prevent the effects of alcohol on the newborn
	[6]	M. Anne George et Al. 2014	Canada	Observational study	A study of the needs of practitioners involved in the management of FASD in British Columbia FASD was a preventable health problem affecting approximately 10% of the population Canadian Guidelines for Diagnosis and Assessment Provincial Outreach Program for Fetal Alcohol Spectrum Disorder (POPFASD)
	[28]	Floyd et al. 2007	USA	Non-randomised retrospective comparative study	Study of 830 women at risk. Half received simple information on the risks of drinking during pregnancy, the other half received motivational talks (4 counseling sessions), a contraceptive consultation, a visit to hospital services (CHOICES project). Intervention goal: to encourage women to change either of the target behaviors (risky alcohol use and ineffective contraception) Significant difference in reduction of FASD for the group receiving counseling
	[29]	George MA et al. 2007	Canada	Action research	The goal was to assist in the prevention of FASD through a participatory research approach involving local women and health workers in developing culturally appropriate methods to help women reduce their alcohol use during pregnancy Designed four different models of culturally appropriate community-based interventions based on five key features: identification of at-risk women; assessment of the woman's substance use; provision of information to women; a delivery method that facilitates the decision to adopt healthier behaviors; and ways to monitor change
	[30]	Peadon et al. 2007	Australie	Recommendation	Identify the role of the general practitioner in the prevention and management of FASD Most health professionals have limited knowledge of FASD and lack confidence in diagnosing and managing children with FASD. General practitioners have an important role to play in identifying women and children at risk of alcohol-related harm and in arranging for referral for assessment and management when necessary
	[31]	Mendoza R et al. 2020	Espagne	Retrospective cross-sectional descriptive study	To analyze the extent to which pregnant women recalled receiving health advice about drinking during pregnancy, what the perceived message was, and whether there was a social inequity 43% of women surveyed reported that they had not received any health advice on this topic. Only 43.5% of the sample recalled receiving the correct message (not to drink alcohol at all during pregnancy) from their midwife, 25% from their obstetrician, and 20.3% from their general practitioner. Women with low levels of education reported the least amount of health advice on this topic The recommended health advice to avoid alcohol consumption during pregnancy did not effectively reach a large proportion of pregnant women
	[32]	Crawford-Williams F et al. 2015	Australie	Qualitative study	Health professionals demonstrated adequate knowledge that alcohol can cause lifelong physical and mental difficulties. However, knowledge of the term FASD was limited. Many did not incorporate the prevention message into their practice, and several questionable judgments were noted It is important to ensure that national guidelines are supported by health professionals
SCREENING	[18]	Loock et al. 2005	Canada	Expert recommendations	Recommended screening tools: CRAFFT (for adolescents) (sensitivity 70%, specificity 94%) and CAGE (T-ACE (sensitivity 70%, specificity 85%) and TWEAK for all women (sensitivity 79%, specificity 83%)) Criteria for referral to expert centers
	[21]	Zoorob et al. 2014	USA	Review of the literature	Primary care practitioners are ideally positioned to screen patients with alcohol use disorders. SBI can improve patient health, reduce alcohol dependence, and prevent future alcohol-exposed pregnancies
	[19]	R.L Floyd et Al. 1999	USA	Expert opinion	The Changing High risk alcOhol use and Increasing Contraception Effectiveness Study (CHOICES). Goal: to identify women at high risk of having an alcohol-exposed pregnancy before they become pregnant, to provide an alcohol risk reduction intervention and to delay pregnancy until alcohol withdrawal Need for multidisciplinary support including primary care
	[33]	SK Clarren et Al. 1998	USA	Retrospective study	Primary prevention essential in FASD Women with FAS children = prime targets (FASD often under-diagnosed) University of Washington: implementation of a screening tool: Fetal Alcohol Syndrome Diagnostic and Prevention Network
	[22]	P M Davis et Al. 2008	Canada	Retrospective study	Primary care practitioners play a key role in screening women at risk for FASD Primary care practitioners may find screening difficult, time-consuming and even uncomfortable Online questionnaire to assess the needs of primary care practitioners in screening
	[34]	Tan CH et al. 2016	USA	Cross-sectional study	The U.S. Preventive Services Task Force (USPSTF) recommended that primary care professionals screen all adults and conduct brief counseling interventions with those who misuse alcohol. The USPSTF preferred the use of three screening tools that measure alcohol use (Alcohol Use Disorders Identification Test, Alcohol Use Disorders Identification Test-Consumption, and National Institute on Alcohol Abuse and Alcoholism Single Question) because these tools detect the full spectrum of alcohol abuse in adults The objective was to estimate the prevalence of alcohol misuse screening practices by primary care professionals and examined factors associated with the use of a USPSTF preferred screening tool A cross-sectional study was conducted on self-reported 2016 DocStyles data from 1,506 primary care providers 96% of providers had reported screening their patients for alcohol abuse. Of those who screened, 38% used a USPSTF recommended screening tool. Provider specialty, knowledge of USPSTF guidelines, and mode of administration of the screening tool were associated with use of a preferred screening tool. About two-thirds did not use a tool capable of detecting FASD
	[35]	O’Connor MJ et al. 2014	Afrique du sud	Intervention	Training community workers to screen for FASD Community workers in Cape Town, South Africa, were trained to screen for FASD in 139 children aged 18 months with prenatal alcohol exposure (PAE). Children were assessed for salient characteristics of PAE subjects using height, weight, head circumference (OFC), philtrum, and lip measurements according to criteria established by the Institute of Medicine. Children who screened positive were referred for diagnostic evaluation to a pediatrician trained in the diagnosis of FASD Of the screen-positive children, 93% were diagnosed with FASD, suggesting that the screening procedure was highly sensitive. Diagnoses included 15% FAS, 23% partial FAS, and 62% ARND The use of community workers to screen for FASD is a promising approach for effective diagnosis of children affected by PAE in areas lacking adequate medical resources
	[36]	Shanley DC et al. 2019	Australie	Mixed intervention	The Yapatjarrathati (named by the local First Nations community and meaning "to be well") project is a mixed-methods implementation trial of a multi-level assessment process to identify FASD in a remote Australian community A culturally sensitive, multi-level neurodevelopmental assessment process to identify FASD and training materials to enhance the skills of remote practitioners with varying levels of expertise were implemented
	[37]	Farlane et al. 2007	Canada	historical article	The article describes the history of the Lakeland Centre for FASD in developing the model and the diagnostic process used to diagnose children and adults. Rural adaptations of similar urban models are discussed. Essential elements of rural in-kind services are also discussed, as well as current challenges. Given the evolution of terminology over the years covered by this article, the term FASD (Fetal Alcohol Spectrum Disorder) is used throughout
	[38]	Washio Y 2017	USA	Intervention	Community-based pilot program to reduce alcohol use among pregnant mothers Participants were required to provide daily breath samples with monetary incentives for negative samples for alcohol. The program has treated four pregnant mothers to date, with an average compliance rate of 94% and no positive alcohol breath samples. Planned future adjustments include the use of a remote reloadable debit card to reinforce daily sample submission, a shift to fully randomized testing programs to avoid false negative results, and expansion of the program's service to additional counties. The community-based program using mobile technology promises to increase opportunities to reinforce a healthy lifestyle during pregnancy
	[22]	Davis PM et al. 2008	Australie	Cross-sectional study	A mail and online survey was distributed in the spring of 2006 to family physicians/general practitioners and nurse practitioners to assess current alcohol risk assessment practices and learning and resource needs among primary health care professionals in Saskatchewan, A total of 876 surveys were distributed and 386 were returned, for an overall response rate of 44.1%. The majority of survey respondents reported rarely or never using a standardized screening tool to assess women's risk for alcoholism or using a less sensitive standardized screening tool. Current practices varied by gender, length of practice, and practice location, while learning and resource needs were more likely to be identified by nurse practitioners, female physicians, and physicians in rural areas. Physicians who had been in practice less than 5 years were more likely to want an online course
	[24]	SC Tough et Al. 2008	Canada	Descriptive article	Important role of primary care practitioners in the prevention and diagnosis of FASD
	[39]	A Hanlon Dearman 2015	Canada	Expert recommendations	Role of the primary care practitioner: screening for child maltreatment, aiming for stability or even placement, educational role to minimize and prevent further maltreatment Role of Primary Health Care Provider (PHCP) in screening children for communication problems in children exposed to alcohol prenatally and referring them early to speech-language pathologists
DIAGNOSIS	[18]	Loock et al. 2005	Canada	Recommandations d’expert	Guide of recommendations for primary care practitioners to enable early diagnosis (and a more favorable evolutionary trajectory) Complex diagnosis facilitated by tools such as the 4-Digit Code
	[39]	A Hanlon Dearman 2015	Canada	Expert recommendations	Important role of primary care practitioners in raising alcohol-related issues Primary care professionals do not routinely bring up alcohol issues (discomfort, lack of time, insufficient remuneration, fear of stigma, and/or lack of information about how to handle a discussion about alcohol use/intervention strategies) Need to have respectful, compassionate conversations; be informative Specific tools: CRAFFT for adolescents (sensitivity 70%, specificity 94%), modified CAGE (T-ACE (sensitivity 70%, specificity 85%) and TWEAK for pregnant and non-pregnant women (sensitivity 79%, specificity 83%)), AUDIT (Alcohol Use Disorder Identification Test) (sensitivity 81%, specificity 95%). Motivational interviewing has its place and is effective in reducing alcohol use during pregnancy Role of primary care professionals in the assessment of children with FASD (referral to care including mental health, information to families) Early diagnosis and treatment of co-morbidities will reduce the burden of care for individuals with FASD and their families. Important role of primary care professionals in early identification of individuals who may have been exposed to alcohol prenatally with a complete medical and prenatal history
	[40]	Wagner B et al. 2018	Australie	Randomised trial	This self-controlled cluster randomized trial evaluated the effectiveness of an 8-week school-based program of the Alert program in improving self-regulation and executive function in children living in remote Australian Aboriginal communities from grades 1 to 6. Trained teachers delivered the Alert program to students in weekly one-hour lessons. Student outcomes were assessed at three different time points. For the intervention condition, data collection took place 2 weeks immediately before and after the intervention, with a subsequent 8-week follow-up. For the control conditions in groups two through four, the control data collection corresponded to the data collection for the intervention condition in the previous group. The primary outcome is change in self-regulation. FASD diagnoses will be determined by review of medical records after data collection is complete
	[41]	V K Temple et Al. 2015	Canada	Descriptive article	An interdisciplinary clinic that diagnoses Fetal Alcohol Spectrum Disorder with a focus on adults. The clinic is part of an interdisciplinary community health agency specializing in intellectual and developmental disabilities
CARE	[39]	Hanlon Dearman 2015	Canada	Expert recommendations	Importance of a multidisciplinary team with the PHCP (Primary Health Care Provider) as the coordinator, focused on the young patient and family Key role of the PHCP and support team in ensuring a successful transition from childhood to adulthood, planning for the resources the child will need in adulthood The PHCP should be aware of support services for individuals with FASD and make early referrals to school and family support services Urgent need for PHCP involvement in the active care of individuals with FASD and their families across the lifespan. PHCPs are trained in screening, prevention and management of health needs PHCP must ensure the transmission of academic knowledge about FASD and its management to families, the educational system (schools…) Role as coordinator of the multidisciplinary care team. The management of FASD involves education, social and justice systems Because specialized care is not always accessible to patients and families in remote areas, the PHCP has an important role as an expert and coordinator of care. The purpose of these guidelines is to provide the PHCP with the latest mental and physical health recommendations for the care of patients with FASD. The PHCP's commitment will provide an integrated system of care for individuals affected by prenatal alcohol exposure and their families
	[20]	Masotti 2003	Canada	Letter to the editor	Major role of primary care in the management of FASD
	[6]	George et Al. 2014	Canada	Analysis	Professional skills program for youth, education program for police officers, social workers and others on how to deal effectively with people with FASD
	[23]	Masotti et Al. 2015	Canada	Expert recommendation	Management of people with FAS: multifactorial (medical and social) with difficulty in the coordination and continuity of care Primary care has a privileged place in the integration of multidisciplinary care This concerns prevention, diagnosis, treatment with an impact on quality of life

## Discussion

The articles agreed that alcohol exposure during pregnancy and its consequences were a major clinical public health problem. Given the front-line role of the general practitioner, they had a primary role in the management of FASD, from prevention to follow-up of affected individuals.

The interventions of the general practitioner were multiple, ranging from prevention (with pregnant women and women of childbearing age), identification, diagnosis, follow-up, education (patient/family/relatives), to coordination of multidisciplinary care (specialized medical, paramedical, social, educational).

The current studies and recommendations were exclusively from English-speaking countries. With regard to this organization, the general practitioner is theoretically the first line of care for people with FASD.

However, several propositions of intervention that can be implemented in general practice have emerged on the basis of the experience of North American and Australian practitioners.

The most relevant screening and diagnostic tools for the prevention and care of patients with fetal alcohol spectrum disorder are grouped together within Additional file 1.

### Prevention and detection

As a front-line actor, the general practitioner has a major role in the primary prevention of fetal alcohol syndrome for pregnant women but also for women of childbearing age and their partners. Exposure to alcohol during the early first trimester is the most harmful period for the baby. Prevention before pregnancy is essential, especially since most pregnancies are discovered after 6 weeks of amenorrhea [19]. Women who had already had a child with FASD were a particularly high-risk population and required special support [20, 21, 33]. Prevention programs have shown their effectiveness [27]. This could involve identifying women at risk of drinking alcohol during pregnancy at an early stage and offering them motivational talks about alcohol consumption and appropriate and effective contraception [19].

Early detection and brief intervention around alcohol consumption is to be favored in the general population, but more particularly among women who want to become pregnant and pregnant women. The same applies to the adult population and the elderly, among whom the prevalence of FASD remains high.

**Fig. 3:**
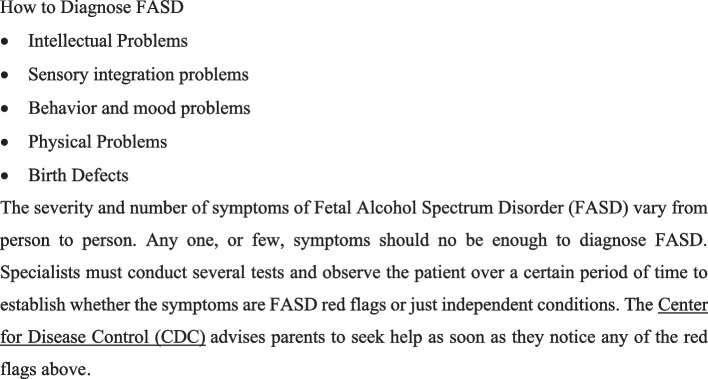
Five Red Flags for FASD

To help with this task, the Fetal Alcohol Spectrum Disorder Resource Center at the University Hospital of La Réunion, in collaboration with the Père Favron Foundation, is raising awareness among general practitioners and other healthcare professionals of the warning signs that may indicate a disorder. Figure 3.

### Diagnosis

Early diagnosis is important to minimize the consequences for the child [39]. To this end, 4 tools are available to assist in the assessment of alcohol consumption: CRAFFT (for adolescents) [18, 39], the "modified CAGE" (T-ACE) [28], the TWEAK [42] for pregnant or nonpregnant women, the AUDIT (Alcohol Use Disorder Identification Test) [39].

The T-ACE is a 4-item questionnaire (Tolerance, Annoyance, Cessation, Awakening) developed specifically for obstetric practice [42]. It deals indirectly with alcohol consumption since it asks about tolerance to the effects of alcohol, the psychological consequences of consumption and the opinion of the entourage on this consumption.

For GP’s daily practice, we would recommend the use of a clinical assessment as promoted within the Canadian Guidelines for diagnosis of FASD across lifespan published in 2016 (update of the 2005 guidelines) incorporating new evidence and improved understanding of FASD diagnosis.

Although from a pathophysiological point of view there are differences between the subtypes of Fetal Alcohol Spectrum Disorder, it turns out that in clinical practice it is difficult for general practitioners to identify these differences.

These tools could be made more accessible for general practitioners using an app or online website.

Even if, as in France, there are recommendations on strategies for identifying FASD, these do not take into account the particularities of the practice of the general practitioner [9]. In the long term, it would be useful to propose a guide of international recommendations for general practitioners to make an early diagnosis possible and thus allow a more favorable evolutionary trajectory [18].

### Follow-up

A good physician–patient relationship is the key to the effectiveness of care for this population, with a global vision of the issues of these patients (socio-economic, communication, family, notion of non-guilt) [20]. The general practitioner must therefore take into account these different determinants to optimize his relationship with the patient (better adherence to follow-up and better therapeutic alliance).

### Education

Therapeutic education is aimed at pregnant women and women of childbearing age [21], at patients with FASD, and at families and close relatives (education concerning alcohol consumption and its impact during pregnancy). Binge drinking is the most common pattern of drinking among pregnant women (more than 4–5 drinks per day) [19]. It results in a spike in blood alcohol levels that have more consequences than the same amount of alcohol consumed over a longer period of time [20]. The general practitioner can assess the risk of binge drinking in pregnant women and women of childbearing age to avoid or reduce its deleterious consequences on the child.

The family has an influential role in modeling adolescent drinking [20]. Special consideration should be given to mothers of children with FASD, as they are at risk of continuing to drink during a new pregnancy [21].

In this same population, the general practitioner must make the family and close relatives aware of the effects of alcohol on pregnancy so that they do not falsely trivialize the consumption of even small amounts of alcohol. This would help to avoid alcohol consumption during pregnancy.

In the case of FASD, the general practitioner should try with the patient's consent to involve the family circle as much as possible: discuss the patient's current situation and involve them in the process of supporting their relative. The family could thus accompany the patient in the care process and support the follow-up.

Education (teaching and awareness) involves all the actors who intervene in the care of the patient with FASD (medical specialists, paramedics including speech therapists [39], police services, social workers) [6], and schools [39]; hence, the coordinating role of general medicine is as follows:

### Role of coordinator

The general practitioner, by virtue of his or her skills and functions in the health care system [43], is the most appropriate person in a patient-centred approach to have a global vision to best coordinate the care pathway of patients and their child with FASD (multi-professional and multi-disciplinary medical/paramedical/social/justice care). It would have the role of expert and informant on the appropriate care system for the patient and family [39].

Extending the reflections of the article [39], screening for FASD risk is not yet systematic in general practice. Physicians cite discomfort in discussing the topic, lack of time, inadequate remuneration, fear of stigma, lack of information on how to conduct a discussion about alcohol use, and lack of knowledge about intervention strategies.

Given these remarks, several ideas could address these limitations:

There is a specific quotation (with a specific remuneration) that could be extended to the management of patients with FASD given the complexity and the need for time to carry out this screening/prevention/diagnosis/coordination/follow-up work. Indeed, the feasibility of such complex quality management over a standard consultation time is not feasible. For example, in France, general personnel consults last 16 min on average according to the *Direction de la recherche des études de l'évaluation et des statistiques* (DREES) of the Ministry of Health [44]. Remuneration in line with the time invested could thus enable doctors to provide quality care to patients with FASD, particularly in regions lacking specialized networks.

In another declarative survey by the DREES [45], 61% of general practitioners indicated that they systematically asked pregnant women about their alcohol consumption, and 77% of general practitioners recommended that their pregnant patients stop drinking altogether during their pregnancy. However, this study showed that 43% considered an occasional drink of alcohol to be an acceptable risk and that for 18%, this level of consumption was safe for pregnancy [45]. In 2017, 65.9% of mothers reported that the physician or midwife who attended them during their last pregnancy informed them of the potential impact of alcohol use on the pregnancy and their child [7]. Only 29.3% of women reported that they had been advised not to drink alcohol during pregnancy [46], yet the American Academy of Family Physicians (AAFP) and the American Congress of Obstetrics and Gynecology (ACOG) recommend that all women of childbearing age be educated about alcohol use to prevent its effects on the newborn [21].

The results of this review raise the question of training physicians during their university and postgraduate courses on the complex management of patients exposed to or suffering from FASD. A better knowledge of the subject would improve their ability to deal with it, promote awareness, and allow for better management (screening / prevention / diagnosis / follow-up / coordination / knowledge of the network). University training is probably currently minimized given the prevalence of this clinical situation.

The Canadian experience of an interdisciplinary clinic for diagnosing FASD is an interesting approach [41]. The establishment of a resource center with a team in charge of referral (university hospitals, medical-psychological centers, medical-psychological-pedagogical centers, medical-social institutions, so-called "ordinary" or specialised schools), coordination and follow-up of the care pathway is probably an appropriate response to the issues of prevention and care of people exposed to or affected by FASD.

The general practitioner ideally has a coordinating role in the management of patients with FASD, but given the current context (lack of time in consultation, lack of visibility of available local actors, lack of specific knowledge on the subject) [39], a single platform would make it possible to assist him/her in the orientation and coordination of the management of these patients.

As was done in Canada in 2008, a qualitative study of the feasibility and acceptability of these interventions could explore the point of view of general practitioners in other countries. Knowing the needs and practices of primary care practitioners would allow for more tailored training for the prevention and identification/screening of alcohol risk in pregnant or childbearing women and their partners.

A cluster randomized trial with a group of primary care practitioners trained in screening for alcohol use during pregnancy versus usual care by untrained primary care practitioners would measure the impact of training on the alcohol use of women of childbearing age and the clinical status of their children born in each group.

The limitations of this literature review are the small number of articles selected, the heterogeneity, and the insufficient level of proof of the studies selected. There are certainly prevention and detection actions that have been evaluated but not published in a scientific journal. Access to grey literature is difficult, which limits the completeness necessary for this literature review.

## Conclusions

The interventions of GPs in the management of patients with FASD are global: prevention, identification, diagnosis, follow-up, education, and the role of coordinator for patients, their families, and pregnant women and their partners.

Prevention interventions would decrease the incidence of FASD, thereby reducing the incidence of mental retardation, developmental delays, and social, educational and legal issues.

GPs would be better equipped and better informed both on the subject and on the networks to optimize the coordination of these complex pathways, while remaining the main interlocutor.

A further study with a cluster randomized trial with a group of primary care practitioners trained in screening for alcohol use during pregnancy would be useful to measure the impact of training on the alcohol use of women of childbearing age and on the clinical status of their children.

### Practical implications

This scoping review highlights the available options for interventions of general practitioners from a global perspective, indicating the importance of clinical tools for everyday practice.

### Supplementary Information

**Supplementary material.**

## Data Availability

As this is a scoping review, we do not have a personal database. The datasets used and/or analysed during the current study available from the corresponding author on reasonable request.

## Abbreviations

AAFP: American Academy of Family Physicians
ACOG: American Congress of Obstetrics and Gynecology
DREES: Direction de la recherche des études de l'évaluation et des statistiques
DSM-5: Diagnostic and Statistical Manual of Mental Disorders V
FAS: Fetal Alcohol Syndrome
FASD: Fetal Alcohol Spectrum Disorder
GP: General practitioner
IOM: Institute of Medicine
ICD: International Classification of Diseases code
ND-PAE: Neurobehavioral disorder associated with prenatal alcohol exposure
PAE: Prenatal alcohol exposure
