# Supplementary material for: Scoping review on the role of the family doctor in the prevention and care of patients with foetal alcohol spectrum disorder
Source: BMC Prim Care. 2024 Feb 22;25:66. doi: 10.1186/s12875-024-02291-x (PMC10882789; doi:10.1186/s12875-024-02291-x)
Supplement: Supplementary file 1 — Supplementary material. [file 12875_2024_2291_MOESM1_ESM.docx]

Supplementary material

Screening and diagnostic tools for prevention and care of patients with fetal alcohol spectrum disorder for general praticionners’ everyday practice

Screening questionnaire for diagnosing problem drinking in adolescent women: CRAFFT*(17)

*(adapted from Knight JR, Shrier LA, Bravender TD, Farrell M, Vander Bilt J, Shaffer HJ. A new brief screen for adolescent substance abuse. Arch Pediatr Adolesc Med. 1999;****153****: 591–6.)*

C Have you ever ridden in a CAR driven by someone (including yourself) who was high or had been using alcohol or drugs?

R Do you ever use alcohol or drugs to RELAX, feel better about yourself, or fit in?

A Do you ever use alcohol or drugs while you are by yourself, ALONE?

F Do you ever FORGET things you did while using alcohol or drugs?

F Does your family or FRIENDS ever tell you that you should cut down on your drinking or drug use?

T Have you ever gotten into TROUBLE while you were using alcohol or drugs?

*Each question on the CRAFFT list is given a score of 1. A cut point of 2 provides moderate sensitivity (70%) and excellent specificity (94%) for identifying alcohol use disorders in adolescents. Any positive answer on the CRAFFT list requires further assessment.

THE T-ACE

T-ACE is a 4 question tool that is a significant indicator of alcohol risk (amount of alcohol sufficient to cause fetal harm). Score from 0 to 5. The value of each question is totaled to determine the final T-ACE score.

T= Tolerance

How many drinks does it take for you to feel the effects of alcohol?

(1 drink of alcohol = 10cl wine, 25cl beer, 2.5cl liqueur/whiskey/pastis)

Binge drinking: consuming 5 or more drinks on the same occasion.

A= Annoyance

Has anyone close to you or a health care professional ever been concerned about your drinking?

C= Cessation

Have you ever tried to reduce your drinking?

E= Awakening

Have you ever needed to drink alcohol in the morning to feel good?

T = 2 points if more than 2 drinks are needed; 1 point if 1 or 2 are needed.

A, C and E = 1 point if yes.

Risk present from 2 points (1 point for some authors)

Above 2 points: high risk.

TWEAK

1) How many drinks do you have to have to feel the first effects of alcohol?

2) How many drinks do you have to have to fall asleep or get drunk? Or if you don't drink to intoxication, what is the most you can drink?

3°) Have the people around you made any remarks about your drinking in the past year?

4°) Have you ever needed alcohol in the morning to feel good?

5°) Have you ever had a drink and then not remembered what you said or did?

6°) Have you ever felt the need to reduce your consumption of alcoholic beverages?

Questions 1 and 2: 3 or more drinks = 2 points / Question 3: "yes" = 2 points / Question 4: "yes" = 1 point

Question 5: "yes" = 1 point / Question 5: "yes" = 1 point / Question 6: "yes" = 1 point

A 2-point score indicates a risk of an alcohol-related problem.

The AUDIT

*(adapted from Chung T, Colby SM, Barnett NP, Rohsenow DJ, Spirito A, Monti PM. Screening adolescents for problem drinking: performance of brief screens against DSM-IV alcohol diagnoses. J Stud Alcohol. 2000;****61****: 579–87.)*


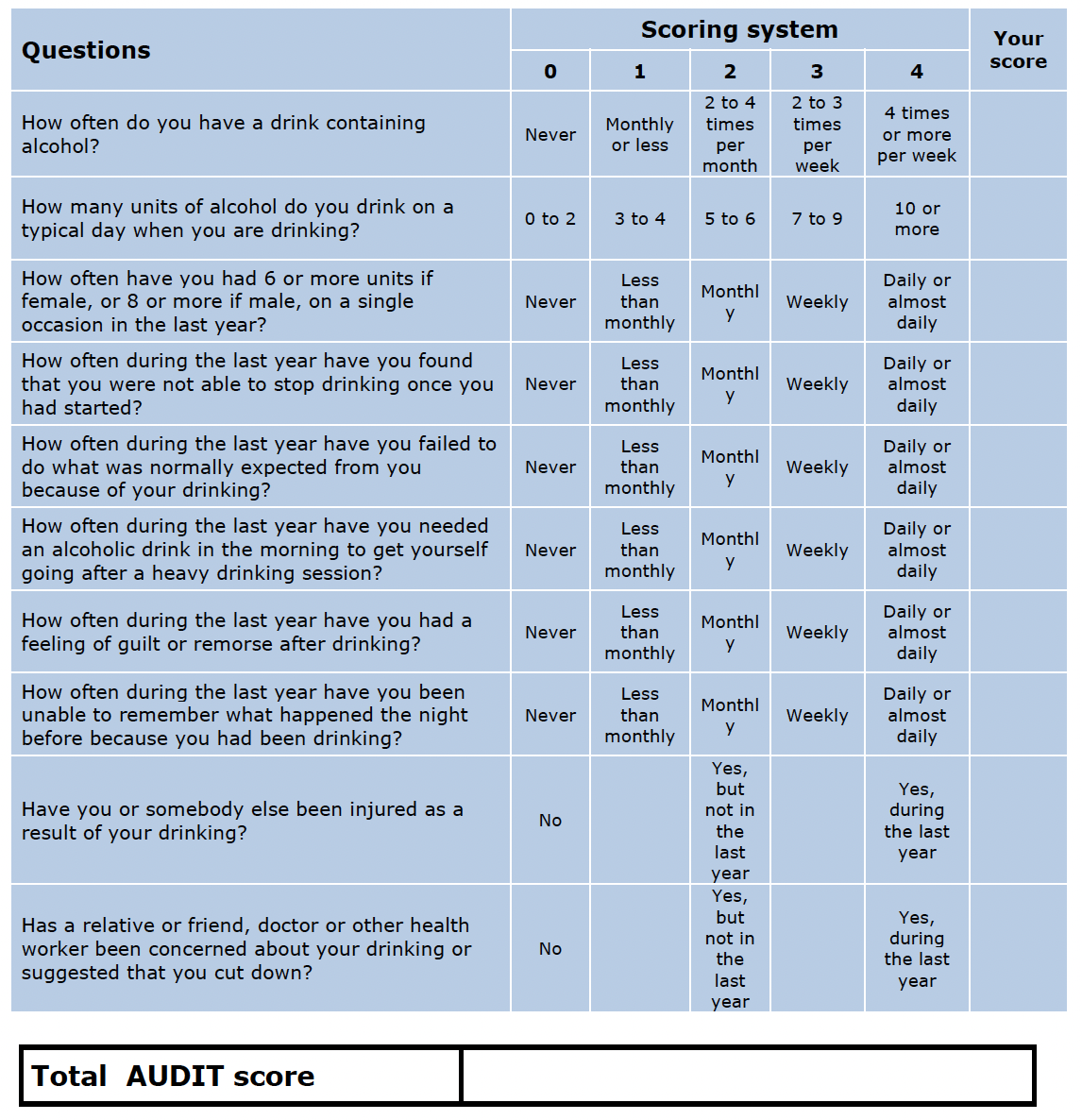


T-ARP

1. How many drinks do you have to have to feel the effect of alcohol?

2. Have people ever annoyed you by criticizing your drinking?

3. Have you ever felt like you should cut down on your drinking?

4. Have you ever had a drink when you got up to calm your nerves or get rid of a "hangover"?

Positive result from two or more YESes (risk of alcohol use during pregnancy)

The 4-Digit Diagnostic Code Grid

|  |  |  |  | 3 | 4 | 4 |  | 4 |  |  |
| --- | --- | --- | --- | --- | --- | --- | --- | --- | --- | --- |
| Severe | Severe | Definite | (4) |  | **x** | **x** |  | **x** | (4) | Hgh risk |
| Moderate | Moderate | Probable | (3) | **x** |  |  |  |  | (3) | Some risk |
| Mild | Mild | Possible | (2) |  |  |  |  |  | (2) | Unknown |
| None | None | Unlikely | (1) |  |  |  |  |  | (1) | No risk |
| **Growth deficiency** | **FAS Facial features** | **CNS Damage** |  | Growth | Face | CNS |  | Alcohol |  | **Prenatal alcohol** |
